# Psychophysiological modelling and the measurement of fear conditioning

**DOI:** 10.1016/j.brat.2020.103576

**Published:** 2020-04

**Authors:** Dominik R. Bach, Filip Melinscak

**Affiliations:** a Wellcome Centre for Human Neuroimaging, Max Planck UCL Centre for Computational Psychiatry and Ageing Research, University College London, 10-12 Russell Square, London, WC1B 5EH, United Kingdom; b Computational Psychiatry Research, Department of Psychiatry, Psychotherapy, and Psychosomatics, University of Zurich, Lenggstrasse 31, 8032, Zurich, Switzerland

**Keywords:** Threat conditioning, Aversive learning, Return of fear, Reconsolidation, Anxiety disorder, Retrodictive validity

## Abstract

Quantification of fear conditioning is paramount to many clinical and translational studies on aversive learning. Various measures of fear conditioning co-exist, including different observables and different methods of pre-processing. Here, we first argue that low measurement error is a rational desideratum for any measurement technique. We then show that measurement error can be approximated in benchmark experiments by how closely intended fear memory relates to measured fear memory, a quantity that we term retrodictive validity. From this perspective, we discuss different approaches commonly used to quantify fear conditioning. One of these is psychophysiological modelling (PsPM). This builds on a measurement model that describes how a psychological variable, such as fear memory, influences a physiological measure. This model is statistically inverted to estimate the most likely value of the psychological variable, given the measured data. We review existing PsPMs for skin conductance, pupil size, heart period, respiration, and startle eye-blink. We illustrate the benefit of PsPMs in terms of retrodictive validity and translate this into sample size required to achieve a desired level of statistical power. This sample size can differ up to a factor of three between different observables, and between the best, and the current standard, data pre-processing methods.

## Highlights

• Fear conditioning studies require quantification of fear learning and memory.
• Statistical power can be increased by increasing measurement precision.
• Measurement precision can be approximated by retrodictive validity.
• Psychophysiological modelling relies on explicit measurement models and offers high retrodictive validity.
• Required sample sizes for a given study can differ by a factor of three, depending on the measurement and analysis method.

## 1 Introduction

Fear conditioning and the ensuing fear memory can be conceptualized as a psychological construct, or in neurobiological terms as a synaptic process, and neither of these is directly observable in humans (or indeed, in most non-human experiments). Thus the quantification of fear conditioning is usually achieved by measuring motor behavior, autonomic nervous system responses, or verbal reports, during or after presentation of a conditioned stimulus (CS): for example, freezing in rodents (Wotjak, 2019), verbal reports and sweating in humans (Boucsein, 2012), as well as bradycardia (Castegnetti et al., 2016) and increased startle response (Brown, Kalish, & Faber, 1951) across many mammals including humans. Although these different observables are sometimes used interchangeably, they differ in several important ways (see (Ojala & Bach, 2019) for a comprehensive review). One is that they may not all relate to the same underlying psychological or synaptic process. For example, lesion studies suggest that verbal report of contingency knowledge depends on hippocampal plasticity, while skin conductance responses (SCR) may depend on amygdala plasticity (Bechara et al., 1995). Next, plasticity-altering drugs appear to have differential impact on SCR and on fear-potentiated startle (Bach, Tzovara, & Vunder, 2018; Soeter & Kindt, 2010). Finally, SCR and pupil size have been suggested to index different components of the same learning process (Tzovara, Korn, & Bach, 2018). Assuming that several observables relate to the same learning process, they may be imbued with different levels of measurement error. For example, fear-potentiated startle appears to differentiate CS+/CS- with much higher effect size than SCR in a retention test without reinforcement (Khemka, Tzovara, Gerster, Quednow, & Bach, 2017). Finally, it turns out that even the same observable can be quantified in a surprising variety of ways, even for rather specific constructs: for example, 16 different methods of quantifying extinction retention from SCR have been identified in the literature (Lonsdorf, Merz, & Fullana, 2019). In some circumstances, different quantification methods for the same observable engender substantially different levels of measurement error (Staib, Castegnetti, & Bach, 2015).

For any researcher planning a clinical or translational fear conditioning study, this raises the question: what is the “best” observable among many different ones, and what is the “best” quantification for this observable? In this article, we focus on a situation where different observables, or quantifications thereof, can be assumed to index the same learning process. In this case, “best” may refer to low measurement error in inferring true scores (translating into high statistical power), low complexity of the measurement system (to be applicable in clinical settings), low financial cost, or a mixture of these. These different criteria can be interrelated. For example, to achieve a desired level of statistical power, a researcher may opt for a low-cost observable with high measurement error (therefore requiring many subjects that also cost money) or a high-cost measure with low measurement error (therefore requiring few subjects).

In this paper, we first discuss a formal framework to answer such statistical and economic questions. This retrodictive validity framework allows selecting measurement methods based on their measurement error. We then present a suite of methods that may have potential to reduce measurement error. This approach, which we have termed psychophysiological modelling, engenders various mathematical (biophysical or descriptive) measurement models, some of which are relevant to human fear conditioning research (Bach et al., 2018; Bach & Friston, 2013). Most of these models are implemented in the open-source toolbox PsPM; some others are available in the toolboxes Ledalab (Benedek & Kaernbach, 2010b), cvxEDA (Greco, Valenza, Lanata, Scilingo, & Citi, 2015) and Pupil (Hoeks and Levelt, 1993), but were developed primarily for applications outside fear conditioning.

## 2 Why do we need this?

From a clinical perspective, where the goal is to help patients, it may appear that the reduction of measurement error provides only incremental benefit, not offsetting the investment. Here, we argue that reduction of measurement error offers a route towards standardization, and in order to increase statistical power may be preferable over large sample size. The benefit of standardisation has been extensively covered in previous reviews (e.g. (Tackett, Brandes, King, & Markon, 2019); and recent work has highlighted how minor changes in data preprocessing can have major impact on conclusions drawn from a particular data set (Lonsdorf et al., 2019; Silberzahn et al., 2018; Steegen, Tuerlinckx, Gelman, & Vanpaemel, 2016). Statistical power, on the other hand, is a function of sample size and effect size. Sample size is often constrained by resources, and some patient groups are difficult to come by, limiting how much one can increase statistical power by increasing sample size. Also, increasing sample sizes incurs costs for each study, whereas increasing effect size by adopting a different measure requires adoption cost only once, and after that confers benefits in perpetuity. Furthermore, increasing sample sizes can be done only prospectively, whereas improved measures can potentially be applied retrospectively to existing datasets. All of this motivates increasing effect size rather than sample size. Effect size is a function of the effect magnitude and its variance, with the variance being determined by inter-individual variation of effect magnitude, and measurement error. In our case, measurement error is composed of inaccuracy and imprecision in quantifying the true fear memory (encompassing, but not restricted to, the measurement error of the recording device). For a particular experimental intervention, the effect magnitude and its inter-individual variation are usually not under experimental control, but one can still attempt to maximize statistical power by minimizing measurement error. Increasing statistical power is likely to yield tangible benefits for patients. Low statistical power not only means that true population effects are more likely to go undetected, but also that significant effects are less likely to indicate true population effects (Button et al., 2013; Ioannidis, 2005) and that reported effect sizes will, on average, be larger than the true population values (Loken & Gelman, 2017). This could be of importance for etiological and translational research. For example, Duits et al. (Duits et al., 2015) conducted a meta-analysis of 44 individual fear conditioning studies including 963 anxiety patients and 1222 healthy persons. Despite the very large sample, significant results are weak and incompatible (see for detailed discussion (Fullana et al., 2019)). Standardising measurement models between the studies that went into the meta-analysis, and increasing statistical power of the measurement models, is one possible way of achieving clearer conclusions. Notably, the application of new measurement models to existing data can also be applied post-hoc, as long as the original psychophysiological signals are available. This means one could increase statistical power of existing data sets in a mega-analysis. A translational example for inconsistent findings concerns the reminder/extinction intervention to reduce fear memory (Schiller et al., 2010). Replication studies were mixed, with a similar number of studies replicating these findings, or not replicating them (Kredlow, Unger, & Otto, 2016). While a meta-analysis suggested an overall effect of the reminder manipulation (Kredlow et al., 2016), an influence of - as yet unknown - boundary conditions is discussed (Lee, Nader, & Schiller, 2017; Phelps & Hofmann, 2019). Investigating these boundary conditions with high-error measures will require large sample sizes; reducing measurement error could potentially reduce the cost associated with these studies and thereby accelerate the translation into clinical application. Also here, mega-analysis of existing data sets with improved analysis methods is possible and may be useful.

## 3 Retrodictive validity: assessing the “goodness” of a measure

Measurement error cannot be precisely quantified because the true scores of a psychological variable are unknown. To overcome this problem, we have suggested using calibration experiments to influence a psychological variable (e.g. fear memory) and compare its intended values (as surrogate “true” values) with their reconstruction from the physiological measurement, for example SCR (Bach et al., 2018; Bach & Friston, 2013). We term this approach “retrodictive validity” as it attempts to retrodict the (intended) values of the experimental manipulation. (Note we have previously used the term “predictive validity”, which however has a potential for confusion (Cronbach & Meehl, 1955)). For example, one could use a very simple fear conditioning experiment with CS+ and CS- that are perceptually easy to distinguish, and assume that most participants will learn the difference between CS+ and CS-. In this dichotomous case, the effect size (e.g. Cohen's d) of the difference between CS+ and CS- in an SCR measure constitutes this measure's retrodictive validity, i.e. it quantifies how “good” that SCR measure is. While there is certainly between-subjects variation in fear memory (effect magnitude) across participants, this aberration of the true scores from the intended values is constant, i.e. independent of the data pre-processing method. Therefore, tuning the pre-processing strategy to increase retrodictive validity is likely to decrease the measurement error. We refer the reader to Bach, Melinscak, Fleming, and Voelkle (2020) for a formal derivation of the statistical method, and the boundary conditions of its applicability.

This approach now allows choosing the data-preprocessing method that yields the highest effect size in an independent benchmark experiment, or optimising the pre-processing method to this end (Bach et al., 2018). To the extent that different observables (e.g. startle eye blink and pupil size responses) index the same learning component, it may also help choosing between observables. Furthermore, it allows more meaningful power analyses than what is current standard practice. Many power analyses in clinical intervention studies are based on effect size estimates from (small) pilot intervention trials. However, effect size estimates from small samples tend to be biased towards larger values, compared to the true population values (Button et al., 2013). Basing power calculations for validation studies on the reported effect size estimates from pilot samples will lead to underpowered studies. However, as pointed out above, the effect size of an intervention on a fear memory measure is determined by the effect size of the intervention on fear memory, and measurement error. Given a particular measurement error, the effect size takes its largest possible value when the effect of the intervention has no variability. This puts an upper limit on possibly measurable intervention effect sizes, and this upper limit is easy to establish by estimating the retrodictive validity of a measure in a control sample (ie. without intervention) (Bach et al., 2018). This then establishes a lower limit on sample sizes required to test an intervention. It turns out that even this lower sample size limit can be higher than what is standard in the field of translational intervention research. For example, a power analysis in Bach, Tzovara, et al. (2018) revealed that to achieve 80% power for detecting an at least 50% reduction of fear memory in a retention test after a placebo-controlled drug intervention, at least N = 74 participants would be required if fear memory is measured by fear-potentiated startle. According to a benchmark experiment with non-optimized hardware (Khemka et al., 2017), N = 156 participants would be required, and using SCR under the same experimental circumstances and with the best available pre-processing strategy would already require a sample size of N = 470 (Khemka et al., 2017). While these numbers are based on effect size estimates from relatively small samples under particular experimental settings, and their numerical values may not generalise, their differences illustrate how small changes in data collection and pre-processing can have dramatic economic consequences in terms of the funds required to run a study. For a given laboratory, it is straightforward to calculate what the additional number of participants will cost, and weigh this against the cost of an alternative measurement system, or the switch to a different data pre-processing strategy. We refer the reader to Bach et al., 2018a, Bach et al., 2018b, Bach et al., 2020 for methodological detail.

## 4 Measurement models

The observables used to infer fear memory usually provide continuous data time series, such as SCR, ECG, or pupil size recordings. There are two principled ways how such time series can be statistically analysed. The first is to enter data from all time bins either into one statistical model, or into a series of independent statistical models, and correct results for the dependency of data points, or for the number of statistical tests, respectively. This procedure is useful when the temporal profile of the observable is unknown or highly variable. However, when for example analysing SCR, many researchers would regard this approach of using correlated data from many time bins as inefficient and impractical. Instead, in most published papers, conditioned SCR time series are condensed into one number per trial, for example SCR peak amplitude during a specified time window, usually combined with criteria on onset and ascent of the SCR. The resulting response estimate is often further summarised, for example by averaging over trials, and subtracting CS- from CS+ responses. In discriminative fear conditioning, the paired difference between CS+ and CS- is often implicitly treated as a measure of fear memory expression.

This process of compressing a continuous data time series into a few small numbers is an example for a heuristic measurement model. This measurement model summarises accumulated expert knowledge into recommendations for data pre-processing, often in the form of publication guidelines (e.g. Blumenthal et al., 2005; Boucsein et al., 2012). For SCR, such guidelines specify, among other things, possible shapes and latencies of CS-evoked SCR. They implicitly assume that CS+ will lead to activity in the sudomotor nerve with higher frequency, or involving more neurons, compared to CS-, and thus engender higher SCR amplitude.

In the following, we explore an alternative to this heuristic approach: formalising accumulated expert knowledge into a mathematically explicit measurement model, and statistically inverting this measurement model. We have termed this approach psychophysiological modelling (Bach & Friston, 2013) and we note that it is similar to measurement models used for fMRI data analysis. Formally related approaches are found across many fields of psychology, for example item-response theory (Embretson & Reise, 2013), expected utility models in behavioural economics (Camerer, 1995), drift-diffusion models in decision psychology (Forstmann, Ratcliff, & Wagenmakers, 2016), or associative learning models (Mathys, Daunizeau, Friston, & Stephan, 2011). Structural equation modelling constitutes an overarching formalism to embed this type of measurement models (Bollen, 1989; Muthén, 2002).

Notably, both the heuristic and the model inversion approach are accessible to optimisation: they can be tuned to maximise retrodictive validity. In principle, it is also possible to create data analysis techniques based on retrodictive validity alone, without any heuristic or mathematical measurement model, in a machine-learning model agnostic to underlying physiology (see e.g. Greco et al., 2017 for a mixed model-based/model-free approach), although the potential of this concept is not clear at the time of writing.

Finally, we note a hybrid approach that was developed specifically for SCR and combines statistical model inversion with heuristic analysis (Alexander et al., 2005; Benedek & Kaernbach, 2010a, 2010b; Greco et al., 2015). Here, a physiological model is statistically inverted to yield the most likely time series of sudomotor nerve firing, given SCR data. In order to infer psychological variables from this estimated neural time series, a heuristic peak-detection approach is used.

All psychophysiological models developed by our laboratory are available in an open-source software, called PsPM (bachlab.org/pspm). We note an additional psychophysiological model that was developed for pupil size analysis (Hoeks & Levelt, 1993); because it is not used in the context of fear conditioning, we refer the reader to Bach et al., 2018a, Bach et al., 2018b for a review.

## 5 Psychophysiological modelling

If the mapping from psychological variable to (time series of) physiological signals (so-called forward model) is fully described, it can be inverted to find the most likely value of the psychological variable, given the measured data (Bach & Friston, 2013). In other words, the value of the psychological variable becomes a parameter in the forward model and is estimated using standard statistical techniques. While some parts of this mapping cannot be fully known (because the psychological variable is unobservable), they can be reasonably well approximated under plausible assumptions. For example, for SCR, the mapping from sudomotor nerve activity to SCR can be empirically investigated with intraneural recordings (we term this “peripheral model”) (Gerster, Namer, Elam, & Bach, 2017). The mapping from psychological variable to neural activity (we term this the “neural model”) can be approximated by replacing the psychological variable with an external stimulus (Bach, Flandin, Friston, & Dolan, 2009; Bach, Flandin, Friston, & Dolan, 2010). The combination of both mappings yields a psychophysiological model (PsPM), which is inverted to infer values of the psychological variable in an experimental context, given the measured physiological data.

In the following, we specifically discuss the different models implemented in PsPM. We note that PsPM encompasses a variety of psychophysiological models, and that there are usually several models for the same observable. These models make different assumptions, which are suitable in particular experimental circumstances (just as there are different peak-scoring procedures). We would generally advocate using a model only in the context of experimental circumstances under which these assumptions are reasonable. For example, the fear conditioning SCR model has been evaluated in paradigms with relatively short CS/US intervals (up to 4 s). The model makes assumptions about plausible sudomotor firing patterns during the CS/US interval, and these may be unrealistic for much longer intervals; hence the model should not be used under these conditions. On the other hand, there is no reason to restrict model use to exactly the same situation as what it has been developed for. For example, the SCR model for fear conditioning has been formally evaluated only for a small number of CS, but there is no reason to suspect that sudomotor activity, or the shape of the SCR, would depend on perceptual features of the CS, and so the basic assumptions appear generalizable to this situation.

In order to create a PsPM, it is first necessary to accumulate knowledge on the forward mapping - the neural and the peripheral model. This is why PsPMs have first been constructed for measures for which the forward mapping was already well-known, such as SCR. Once the forward model is established and mathematically formalised, it can be inverted using standard statistical techniques. The model itself, and the inversion routine, usually contain some parameters or settings that are based on approximations or expert intuition. These are then optimized on independent samples to maximise retrodictive validity. Most PsPMs act on pre-processed (filtered and artefact-reduced) data, and the pre-processing process can be optimized as well (see e.g. Bach, Friston, & Dolan, 2013; Khemka et al., 2017; Staib et al., 2015 for examples).

PsPMs have been designed for estimating various psychological variables from different physiological signals (see Bach et al., 2018 for a comprehensive summary), including models for SCR (Bach, 2014a, 2014b; Bach, Daunizeau, Friston, & Dolan, 2010; Bach, Daunizeau, Kuelzow, Friston, & Dolan, 2011; Bach et al., 2009; Bach, & Flandin et al., 2010; Bach & Friston, 2012, 2013; Bach, Friston, & Dolan, 2010; Bach et al., 2013; Bach & Staib, 2015; Gerster et al., 2017; Staib et al., 2015), pupil size responses (Korn & Bach, 2016; Korn, Staib, Tzovara, Castegnetti, & Bach, 2017), heart period/heart rate changes (Castegnetti et al., 2016; Paulus, Castegnetti, & Bach, 2016), respiratory responses (Bach, Gerster, Tzovara, & Castegnetti, 2016; Castegnetti, Tzovara, Staib, Gerster, & Bach, 2017), and startle eye blink EMG (Khemka et al., 2017). In the following, we give a brief overview of those models that have been used for inferring fear memory in classical fear conditioning tasks.

### 5.1 Skin conductance responses

#### 5.1.1 Background and model

Conditioned stimuli (CS) elicit a SCR, which differs in amplitude between CS+ and CS-, and occurs at some point before the time point of possible unconditioned stimulus (US) delivery (Boucsein, 2012). Because SCR have a long tail (>120 s even for standardly filtered data (Bach, & Flandin et al., 2010)), CS and US responses overlap within and between trials, depending on the experimental timings. PsPM takes this overlap into account. There are two SCR models implemented in PsPM. One (termed “non-linear model”) assumes variable latency, duration and amplitude of a CS-induced, neural input, and estimates these parameters from observed SCR time series (Bach, & Daunizeau et al., 2010). The amplitude of this neural input is assumed to linearly relate to fear memory strength. This model was optimized for experiments with short (up to 4 s) CS-US interval. The other model (termed “GLM”) describes evoked, constant-latency, SCR (Bach, 2014a; Bach et al., 2009; Bach, & Flandin et al., 2010; Bach et al., 2013). It could possibly be used to analyse conditioned and unconditioned responses in fear conditioning experiments, if they can be assumed to have constant latency and duration. In particular for very short CS-US intervals (<2 s), the flexibility of estimating response latency in the non-linear model may not be needed and the increased degrees of freedom may lead to overfitting.

#### 5.1.2 Data pre-processing

In addition to model-based analysis of SCR, the software package PsPM provides quality control and an interface for visual artefact annotation; annotated artefacts are automatically excluded from model inversion.

#### 5.1.3 Evaluation

The non-linear model has been compared with Ledalab and peak scoring by ourselves. This study investigated fear acquisition (ie. under continuous reinforcement) in two experiments with 3.5 s CS-US interval (Staib et al., 2015). In both experiments, PsPM-derived fear conditioning estimates distinguished CS+ and CS- better (i.e. had decisively higher retrodictive validity) than standard peak-scoring, or Ledalab-derived estimates (Benedek & Kaernbach, 2010b). Peak scoring yielded a weighted average effect size of 0.44, Ledalab an effect size of 0.53, and PsPM an effect size of 0.75. For our example of testing a placebo-controlled fear memory intervention with at least 80% power to detect a 50% reduction in fear memory with a one-tailed test, these effect sizes translate into minimum sample sizes of N = 514 for peak-scoring, N = 342 for Ledalab, and N = 174 for PsPM. A different research team evaluated the GLM approach in two fear conditioning experiments with short (4 s) CS-US interval (Green, Kragel, Fecteau, & LaBar, 2014); notably this is a situation in which our previous work suggests that the GLM approach may be suboptimal (Bach, & Daunizeau et al., 2010). This study compared several peak-scoring methods, Ledalab, and PsPM. Different from our own validation, their aim was not to distinguish CS+/CS-. Instead they averaged over CS+/CS- trials and sought to distinguish different experimental phases and conditions. In one experiment, they found that the GLM approach distinguished conditioning/extinction/renewal phases better than all other methods. In another experiment, they sought to distinguish pre-conditioning phase, conditioning phase, and three conditions from a generalization phase. They found that manual peak scoring distinguished these five conditions better than any other method (Green et al., 2014). We note that it is somewhat less clear to what extent these phases and conditions should theoretically be different.

### 5.2 Pupil size responses

#### 5.2.1 Background and model

Pupil size is mainly influenced by ambient luminance, but pupil dilation is also observed in relation to psychological processes (Hoeks & Levelt, 1993; Korn et al., 2017). We have shown, across several experiments, that CS elicit pupil size responses that depend on the type and sensory modality of the CS, but the difference between a response elicited by CS+ and CS- is rather constant across experiments (Korn et al., 2017). This difference is time-locked to the CS, as we have empirically shown for CS/US intervals up to 6 s (Korn et al., 2017). The PsPM for fear-conditioned pupil size responses estimates the magnitude of fear memory strength as amplitude of a neural input into the pupillary system (Korn et al., 2017), using a GLM approach under the assumption of constant response latency. PsPM also implements a model for illuminance responses, which can be used to correct data for pupil size changes induced by luminance changes (Korn & Bach, 2016).

#### 5.2.2 Data pre-processing

For ensuring suitable data quality, PsPM includes an automated artefact correction algorithm that can also combine two pupil measurements into a less noisy one (Kret & Sjak-Shie, 2019), a possibility to account for the pupil foreshortening error due to gaze deviation (Hayes & Petrov, 2016), and features to exclude data points based on loss of fixation. Missing data points due to saccades, blinks, or loss of fixation, are automatically ignored for the model inversion.

#### 5.2.3 Evaluation

In several experiments, the model for fear-conditioned pupil size responses had decisively higher retrodictive validity than peak-scoring (Korn et al., 2017). In this paper, pupil size was measured during fear acquisition (ie. under continuous reinforcement) in five experiments with different CS modalities (auditory, visual, somatosensory). Excluding the first experiment, on which the PsPM was developed, peak-scoring yielded a weighted average effect size of 0.60, and PsPM an effect size of 0.82. For our example of testing a placebo-controlled fear memory intervention with at least 80% power to detect a 50% reduction in fear memory, these effect sizes translate into minimum sample sizes of N = 278 for peak-scoring, and N = 150 for PsPM. Responses to the US can be fully disambiguated from responses to the CS (Korn et al., 2017), such that it is in principle possible to analyse trials with a US, and to analyse the US response itself. We note that there is no independent evaluation of the method as yet.

### 5.3 Fear-conditioned bradycardia

#### 5.3.1 Background and model

CS + elicit a reduction in heart rate, or equivalently an increase in heart period, in many species including humans (see Castegnetti et al., 2016 for a review of studies). Heart period (the reciprocal of heart rate) has been shown to linearly relate to parasympathetic neural input into the heart (Parker, Celler, Potter, & Mccloskey, 1984; Rosenblueth & Simeone, 1934) and is therefore more likely to linearly relate to psychological variables than heart rate (Berntson, Cacioppo, & Quigley, 1995). The model therefore takes heart period as input, which is interpolated between heart beats into a continuous time series (Paulus et al., 2016). We have demonstrated across several experiments that, similar to fear-conditioned pupil size responses, a CS elicits a heart period response with dynamics that depend on the type and sensory modality of the CS, but the difference between responses elicited by CS+ and CS- is rather similar across experiments (Castegnetti et al., 2016). Different from pupil size, this difference occurs with a constant latency before the time point of possible US delivery (Castegnetti et al., 2016, see Castegnetti et al., 2017 for a CS/US interval of 6 s). The PsPM for fear-conditioned bradycardia estimates the magnitude of fear memory strength as amplitude of the neural input (Castegnetti et al., 2016) in a GLM approach that assumes constant latency of the response.

#### 5.3.2 Data-preprocessing

The model takes a continuous, interpolated heart period time series as an input. To create this time series, PsPM includes several algorithms to detect heartbeats from ECG or pulse oximetry data, including an interface for semi-automatic visual inspection, and an interpolation facility that includes quality control for implausible values.

#### 5.3.3 Evaluation

The method was evaluated in four experiments with different CS types (visual, auditory) with 3.5 or 4.0 s CS/US interval during fear acquisition (ie. under continuous reinforcement). There is no standard peak-scoring method available for this type of measurement; our own peak-scoring methods provided decisively worse retrodictive validity than the PsPM-based method (Castegnetti et al., 2016). Due to the lack of an alternative standard method we cannot make strong statements on PsPM's benefit here. Excluding the first experiment, on which the PsPM was developed, PsPM yielded an effect size of 0.97. For our example of testing a placebo-controlled fear memory intervention with at least 80% power to detect a 50% reduction in fear memory, this effect size translates into a minimum sample size of N = 108.

### 5.4 Respiration amplitude responses

#### 5.4.1 Background and model

There is a dearth of studies on phasic respiration changes due to brief stimuli in general, and during fear conditioning in particular (see Van Diest, Bradley, Guerra, Van den Bergh, & Lang, 2009 for a notable exception). Across several experiments, we have shown that CS induce a respiration amplitude response with shape that depends on type and sensory modality of the CS, but that the difference in response to CS+ and to CS- is rather similar and characterised by an early reduction in respiration amplitude and a later increase (Castegnetti et al., 2017). Notably, the model was developed on single-chest belt data. It is therefore not clear yet whether the form of the response curve indicates an overall change in breathing or a shift from thoracic to abdominal breathing and back. Experiments with two chest belts will be required to determine this question (Binks, Banzett, & Duvivier, 2007). There is some indication that the conditioned respiration response occurs with a constant latency before the time point of possible US delivery, although the available evidence is not entirely conclusive (Castegnetti et al., 2017). The PsPM for fear-conditioned respiration amplitude estimates the magnitude of fear memory strength as amplitude of a neural input (Castegnetti et al., 2017).

#### 5.4.2 Data pre-processing

The model takes a continuous, interpolated respiration amplitude time series as an input. To create this time series, PsPM includes algorithms to detect inspiration onset and quantify respiration amplitude from single chest belt measurements that use bellows- or cushion-based systems.

#### 5.4.3 Evaluation

There is no standard peak-scoring method available for this type of measurement; our own peak-scoring methods provided decisively worse retrodictive validity than the PsPM-based method (Castegnetti et al., 2017). Due to the lack of an alternative standard method we cannot make strong claims on PsPM's benefit. Excluding the first experiment, on which the PsPM was developed, PsPM yielded an effect size of 0.61. For our example of testing a placebo-controlled fear memory intervention with at least 80% power to detect a 50% reduction in fear memory, this effect size translates into a minimum sample size of N = 268.

### 5.5 Fear-potentiated startle

#### 5.5.1 Background and model

Loud sounds with sudden onset elicit a motoric startle response in many mammal species, which is supposed to protect the organism from predator attack (Yeomans, Li, Scott, & Frankland, 2002). This startle response is modulated by the presence of a fear-conditioned CS+, a phenomenon termed fear-potentiated startle ((Brown et al., 1951), see for review (Bach, 2015)). In humans, startle response is easily quantified by measuring the activity of the orbicularis oculi muscle using electromyography (Blumenthal et al., 2005). The PsPM for startle eye blink responses estimates latency and amplitude of a (not necessarily modulated) neural input into the startle circuit (Khemka et al., 2017). Fear memory strength is assumed to be the difference between neural input into the startle system during a CS+, and a CS-.

#### 5.5.2 Data pre-processing

The PsPM takes as input data a pre-processed electromyography time series; pre-processing was optimized to enhance retrodictive validity (Khemka et al., 2017) and is implemented in the PsPM toolbox.

#### 5.5.3 Evaluation

There are many different peak-scoring methods available for fear-potentiated startle. We found pronounced differences between PsPM and different peak-scoring approaches in individual experiments, but no consistent benefit of any one method (Khemka et al., 2017). Excluding the first experiment, on which the model was developed, PsPM yielded an effect size of 0.96 in two experiments that did not use optimized hardware. For our example of testing a placebo-controlled fear memory intervention with at least 80% power to detect a 50% reduction in fear memory, this effect size translates into a minimum sample size of N = 110.

## 6 Discussion

High statistical power is desirable for answering a range of clinical and translational questions in fear conditioning research. High statistical power can be achieved by increasing sample size, or by increasing accuracy and precision of a fear memory measure, and its pre-processing. In order to quantify the goodness of a method for measuring fear memory strength, it is necessary to make assumptions. One assumption is that there are experiments that create distinguishable values of fear memory strength. This allows for quantification of how well a method recovers this intended difference, something we term here “retrodictive validity”. Optimising fear conditioning measures to yield high retrodictive validity increases statistical power. As one among several strategies to quantify fear memory, PsPM inverts a statistical model of how fear memory influences physiological processes. PsPMs have been developed for SCR, pupil size, bradycardia, respiration amplitude, and fear-potentiated startle. Several of them offer higher retrodictive validity than automated peak-scoring methods. Crucially, differences in retrodictive validity can translate into substantial differences for minimum required effect sizes, for example to test fear memory interventions. While the absolute numbers we have provided depend on the precise experimental circumstances, their differences are substantial: the sample size for the worst quantification method can be almost three times as large as for the best quantification method. As long as different observables relate to the same learning component, the framework of retrodictive validity also allows to compare different observables against one another: for example, to measure fear memory retention under formal extinction (i.e. in the absence of reinforcement), fear-potentiated startle may offer much higher retrodictive validity than SCR or bradycardia (Khemka et al., 2017). Again, this translates into a minimum required sample size that can be three times as large for one measure as compared to another.

None of these psychophysiological measure provides very large effect sizes to distinguish CS+ and CS-, raising a question whether there are better behavioural assessments of fear memory, beyond declarative memory which may depend on a different learning process (Bechara et al., 1995). To our knowledge, no such measures exist, and we have empirically shown that CS-evoked SCR, pupil size, and bradycardia, provide higher retrodictive validity for inferring fear memory than a measure of Pavlovian-to-instrumental transfer (Xia, Gurkina, & Bach, 2019). This state of affairs could motivate further investment into improving psychophysiological measures. While we generally advocated selecting the most precise measure, it appears, however, that not all psychophysiological measures relate to the same underlying learning process. In particular, it has been suggested that SCR may not relate to US prediction but rather to CS associability (Li, Schiller, Schoenbaum, Phelps, & Daw, 2011; Zhang, Mano, Ganesh, Robbins, & Seymour, 2016), which is higher for CS + than CS- in a partial reinforcement schedule as typically used in human research. Pupil size, on the other hand, may more unambiguously relate to US prediction (Tzovara et al., 2018). This is a possibility that will require further investigation and could guide the choice of fear conditioning measures over and above retrodictive validity considerations.

Furthermore, the psychophysiological modelling approach in general – and the methods implemented in the PsPM toolbox specifically – have been evaluated only in limited experimental circumstances and by a small group of researchers. More methodological research on these and other methods could help establishing a clearer picture on what the best measurement approach is in different research scenarios. A perennial problem for continuous-time measures with long tails is that CS and US (or US omission) responses overlap. Thus, it is important to demonstrate that discriminability of CS+/CS- trials is not due to a confounding impact of US (or US omission) responses (see Bach & Friston, 2012 for an example of such demonstration). Finally, while for some measures such as SCR the forward mapping is well known, relatively simple, and accessible by intraneural recordings, (Boucsein, 2012), for some others such as heart period responses the response dynamics are incompletely understood. Here, in particular CS- responses can be rather variable (Paulus et al., 2016), while the CS+/CS- difference appears more stereotypical (Castegnetti et al., 2016). Such variability can limit the development of suitable PsPMs. We note that this may equally limit heuristic methods, such that independent of the measurement model, more research on “classical” psychophysiological forward mappings appears warranted.

As a limitation of the retrodictive validity criterion in general – and independent of whether one uses PsPM or heuristic analysis strategies – optimized procedures that increase retrodictive validity are optimal in a strict sense only under the same (or similar) experimental procedures used in the benchmark experiment. It may still be possible to generalise them in simple cases such as going from one pair of CS colours in a benchmark experiment to another pair of colours in a substantive experiment, where a body of evidence suggests that this does not substantially alter the shape of a CS-induced SCR. In other cases, such as increasing the complexity of an intra-trial procedure, or the timing of events, this may be more difficult. There are however many applications even of a very strict approach. For example, one can keep a memory retention test exactly equal between the benchmark experiment, and the substantive research, and add “memory editing” (Phelps & Hofmann, 2019) interventions before the test. Under these circumstances, it is likely that the most utile method generalizes from the benchmark to the intervention experiment, as long as it measures the attribute that the memory editing technique is supposed to alter.

With the development of the retrodictive validity criterion and the openly available toolbox PsPM, we hope to encourage clinical and translational researchers to maximise the benefit they can obtain from their fear conditioning measures: by using it, by contributing to it, or by developing alternative high-validity measures. Current developments include novel measurement modalities as well as combinations of different fear memory measures.
